# High-Grade Glioma With Parotid Metastasis: A Case Report of Long-Term Treatment and Follow-Up

**DOI:** 10.7759/cureus.78232

**Published:** 2025-01-30

**Authors:** Raed Abu Jarir, Noman Shah, Muhammad Mohsin Khan, Mhran Daie, Abdulrazzaq Haider, Ali Ayyad

**Affiliations:** 1 Neurosurgery, Hamad General Hospital, Doha, QAT; 2 Pathology and Laboratory Medicine, Hamad General Hospital, Doha, QAT

**Keywords:** astrocytoma grade 4, extracranial metastasis, glioblastoma, high-grade glioma, idh mutation, parotid gland

## Abstract

High-grade gliomas (WHO Grade 4) are aggressive and common primary brain tumors of glial origin with poor prognosis. The classification of high-grade astrocytomas has evolved, improving the distinction between tumor subtypes. Previously, isocitrate dehydrogenase (IDH)-mutant astrocytomas that progressed to Grade 4 were referred to as secondary glioblastomas. They are now categorized as astrocytoma, IDH-mutant, WHO Grade 4, reflecting their unique molecular and clinical features. While intracranial spread is common in high-grade gliomas, extracranial metastases remain exceptionally uncommon. This case report highlights a 31-year-old woman who initially presented with seizures and progressive neurological symptoms. Initial imaging revealed a diffuse astrocytoma (WHO Grade 2), confirmed by histopathology and molecular testing as an IDH mutant. Despite surgery, radiotherapy, and chemotherapy, follow-up imaging demonstrated progression to high-grade glioma (WHO Grade 4) with intracranial tumor growth and, eventually, extracranial metastases to the cervical lymph nodes and parotid gland. Histopathological examination of the metastatic lesions revealed high proliferative activity and molecular studies confirmed specific genetic alterations consistent with the primary tumor. This case underscores the importance of comprehensive clinical evaluation, vigilant imaging follow-up, and advanced molecular diagnostics in high-grade gliomas (WHO Grade 4). It also highlights the rare metastatic behavior of IDH-mutant astrocytomas (previously classified as secondary glioblastomas) and emphasizes the need for long-term monitoring and individualized treatment strategies to improve outcomes.

## Introduction

High-grade gliomas are among the most malignant and common primary brain tumors in adults. It is notorious for its aggressive nature and poor prognosis and is characterized by rapid progression and high mortality [1,2]. The latest World Health Organization (WHO) classification of tumors of the central nervous system (CNS) in 2021 gave more importance to the molecular diagnosis of CNS tumors based on established histology, immunohistochemistry diagnostic criteria, and molecular predominance [2]. Additionally, the 2021 classification replaces Roman numerals with Arabic numerals for tumor grading, with Grade 4 denoting the highest level of malignancy, to reduce ambiguity and improve consistency. In previous CNS classifications, the glioblastoma multiforme is defined as Grade 4 astrocytoma and is divided into either isocitrate dehydrogenase (IDH) mutant or IDH wild; however, since the WHO CNS Classification 2021 update, glioblastoma multiforme is nominated as glioblastoma with IDH wild type, and the other similar histology with IDH mutant variant is called astrocytoma Grade 4 [2]. Notable since the last update, we are no longer using the term multiforme [2].

High-grade glioma predominantly affects the elderly, more often males, with a median survival rate of about 15 months despite standard treatments like surgery, radiotherapy, and chemotherapy [1]. Rapid intracerebral invasion is a hallmark of glioblastoma, but on the other hand, extracranial metastases are extremely rare, occurring in only 0.4-0.5% of patients [3]. This rarity is attributed to glioblastoma's swift intracranial progression, limited overall survival, and the brain's unique environment that is unfavorable for extracranial tumor cell spread but also essential for the vital activity and sustenance of tumor cells [4]. Although the glymphatic system, responsible for waste clearance in the central nervous system, may influence the behavior of tumor cells and their ability to metastasize, its role is not yet fully understood. However, high-grade astrocytoma, IDH-mutant, Grade 4, previously regarded as secondary glioblastomas under earlier WHO classifications, usually develops from a preexisting lower-grade tumor and has a better prognosis than glioblastoma (IDH-wild type by definition).

Recent studies have shown that glioblastoma can metastasize to various extracranial locations, including the bone, lymph nodes, and lungs, with a minority of cases involving the liver, soft tissue, and skin. Cervical lymph node metastases are most common, often corresponding to the craniotomy site [5]. The pathogenesis of glioblastoma metastasis is still a topic of ongoing research, with hypotheses suggesting that tumor cell invasion through the venous system or direct dural penetration, coupled with the breakdown of the blood-brain barrier, and potentially the involvement of the glymphatic system, could ease systemic dissemination.

The case presented here is a striking illustration of high-grade glioma and the potential for extracranial metastasis, highlighting the disease's unpredictable and devastating trajectory. It involves a 31-year-old female initially diagnosed with a WHO Grade II astrocytoma (as per the 2016 WHO classification) that progressed to a Grade 4 IDH-mutant astrocytoma (as per the 2021 WHO classification) in approximately 18 months, ultimately leading to systemic metastasis. This rapid progression and distant metastases are atypical, as extracranial spread is rarely observed in astrocytoma Grade 4 (formerly classified as secondary glioblastoma) cases and is more commonly associated with primary glioblastoma [5].

Considering the aforementioned case and the growing body of evidence, it becomes clear that high-grade glioma, particularly in its rare metastatic form, poses significant diagnostic and therapeutic challenges. In cases of cervical metastasis, surgical intervention is often deferred in favor of chemotherapy and radiotherapy, which may contribute to underreporting and a limited understanding of these rare occurrences. This underscores the importance of vigilant monitoring for systemic involvement in high-grade glioma patients, especially those with secondary glioblastoma (astrocytoma Grade 4), who show longer survival times due to initially favorable histological and molecular patterns. The case also emphasizes the need for a comprehensive understanding of high-grade astrocytoma metastatic potential to improve patient outcomes and develop targeted therapeutic strategies [6].

## Case presentation

A 31-year-old female initially presented in late 2013, approximately two years before her diagnosis, with a four-month history of partial complex seizures. Her medical history included being overweight (body mass index (BMI): 29) and having hypertension for the past year, which was managed with antihypertensive medication, and type II diabetes mellitus was managed with oral hypoglycemic agents and hypothyroidism on replacement therapy. She was not on insulin therapy. Family history revealed breast cancer in her maternal aunt, prostate cancer in her paternal uncle and a brain tumor in a third-degree nephew. This timeline highlights how her diagnosis and progression occurred before the 2016 WHO Classification update when molecular markers like IDH mutation status became pivotal in glioma classification. 

Genetic counseling was not conducted in this case, but the patient’s family history of breast cancer in her maternal aunt, prostate cancer in her paternal uncle, and a brain tumor in a third-degree relative raises the possibility of a hereditary predisposition. Interestingly, IDH mutations, which are central to glioma classification, have also been linked to breast and prostate cancers. While the connection between these cancers and gliomas is not fully understood, it underscores the importance of investigating familial cancer patterns. Genetic counseling in similar cases could help uncover potential predispositions and guide more personalized approaches to treatment.

The patient was fully conscious with a Glasgow Coma Scale (GCS) score of 15 and exhibited no focal neurological deficits. A non-contrast computed tomography (CT) scan and later magnetic resonance imaging (MRI) revealed a 4.7 × 5.5 × 5.3 cm non-enhancing right temporal mass lesion with peri-lesion edema and a midline shift of 1.1 cm. The lesion was hypo-intense on T1, hyper-intense on T2, and had a mixed pattern on fluid-attenuated inversion recovery (FLAIR) imaging. It displayed high choline and low N-acetyl-aspartate (NAA) without diffusion restriction.

First surgery

**Figure 1:**
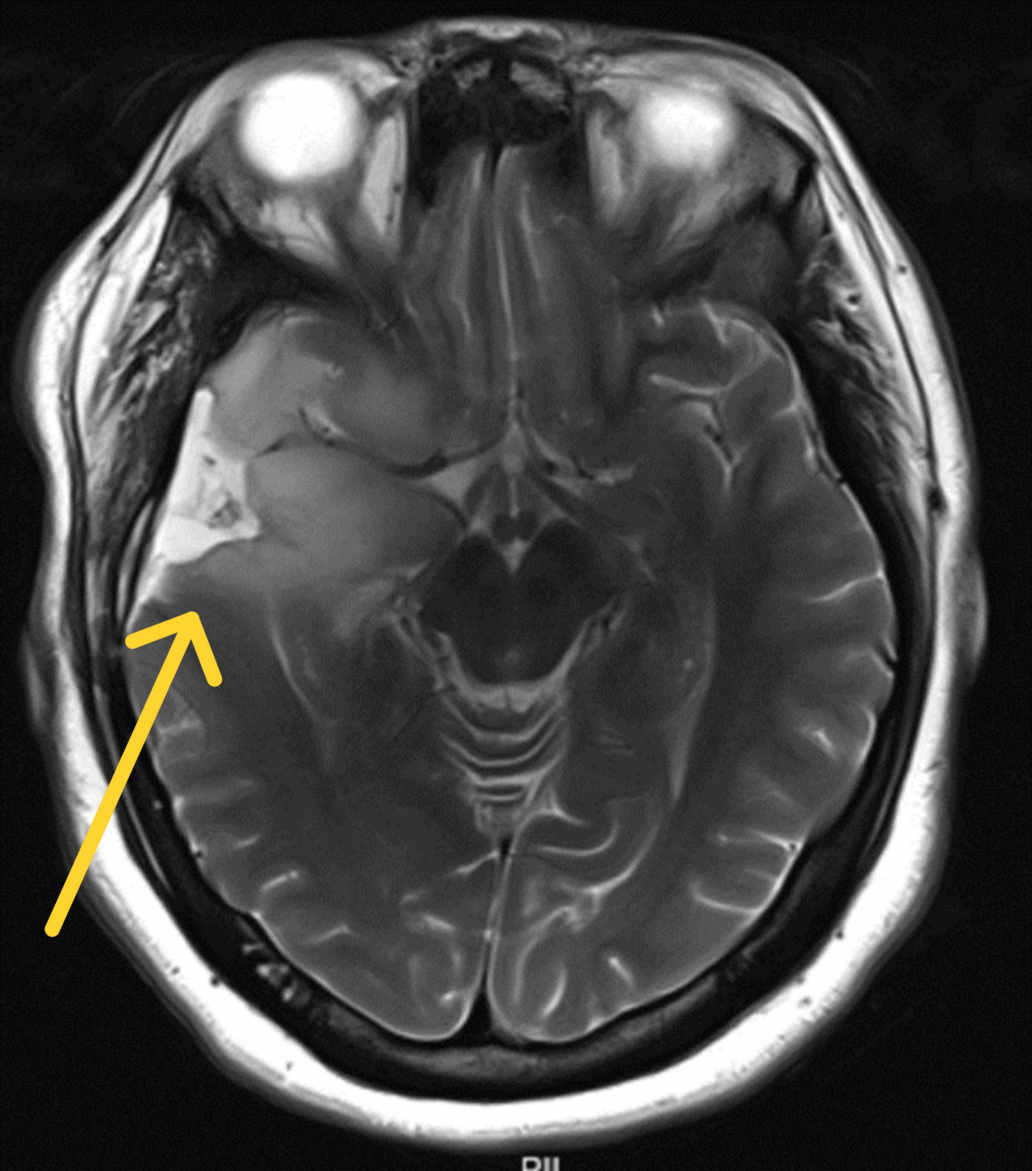
Follow-up MRI approximately 12 months postoperatively (T2-weighted axial cut) showing tumor recurrence in the right temporal lobe with associated edema.

**Figure 2:**
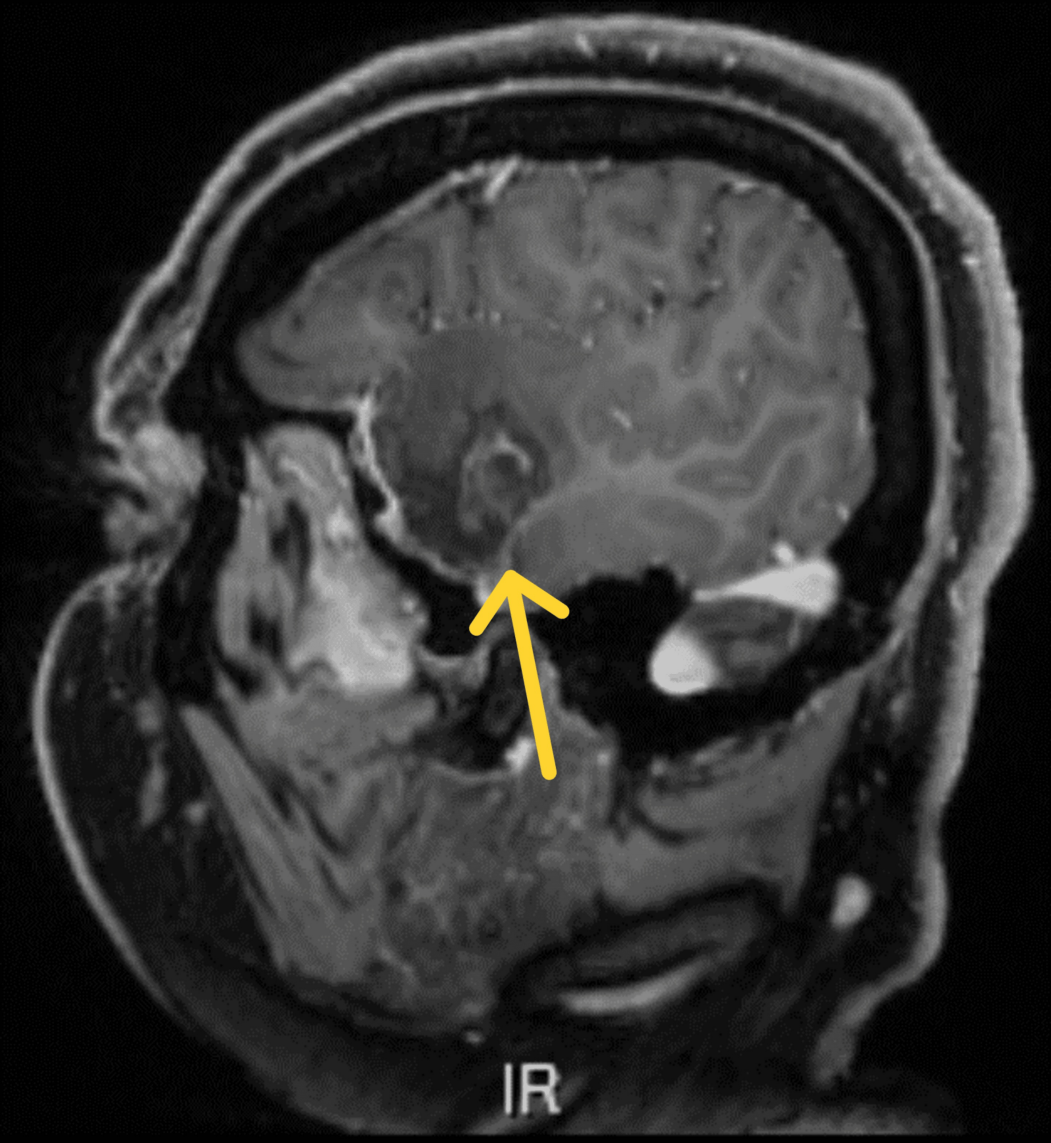
Follow-up MRI approximately 12 months postoperatively (T1-weighted with contrast, sagittal view) showing tumor recurrence in the right temporal lobe.

A craniotomy and tumor debulking with an anterior temporal lobectomy were performed, and gross total resection was achieved. Histopathology studies confirmed the diagnosis of diffuse fibrillary astrocytoma, Grade II. The patient did not receive adjuvant radiotherapy or chemotherapy at this stage. A follow-up MRI approximately 12 months later, after the first surgery, showed tumor regrowth, measuring 6.8 × 6.4 × 5.2 cm. Recurrence and tumor progression was evident on imaging, as shown in Figure 1 (T2 axial cut) and Figure 2 (T1 with contrast, sagittal view).

Follow-up and second surgery

**Figure 3:**
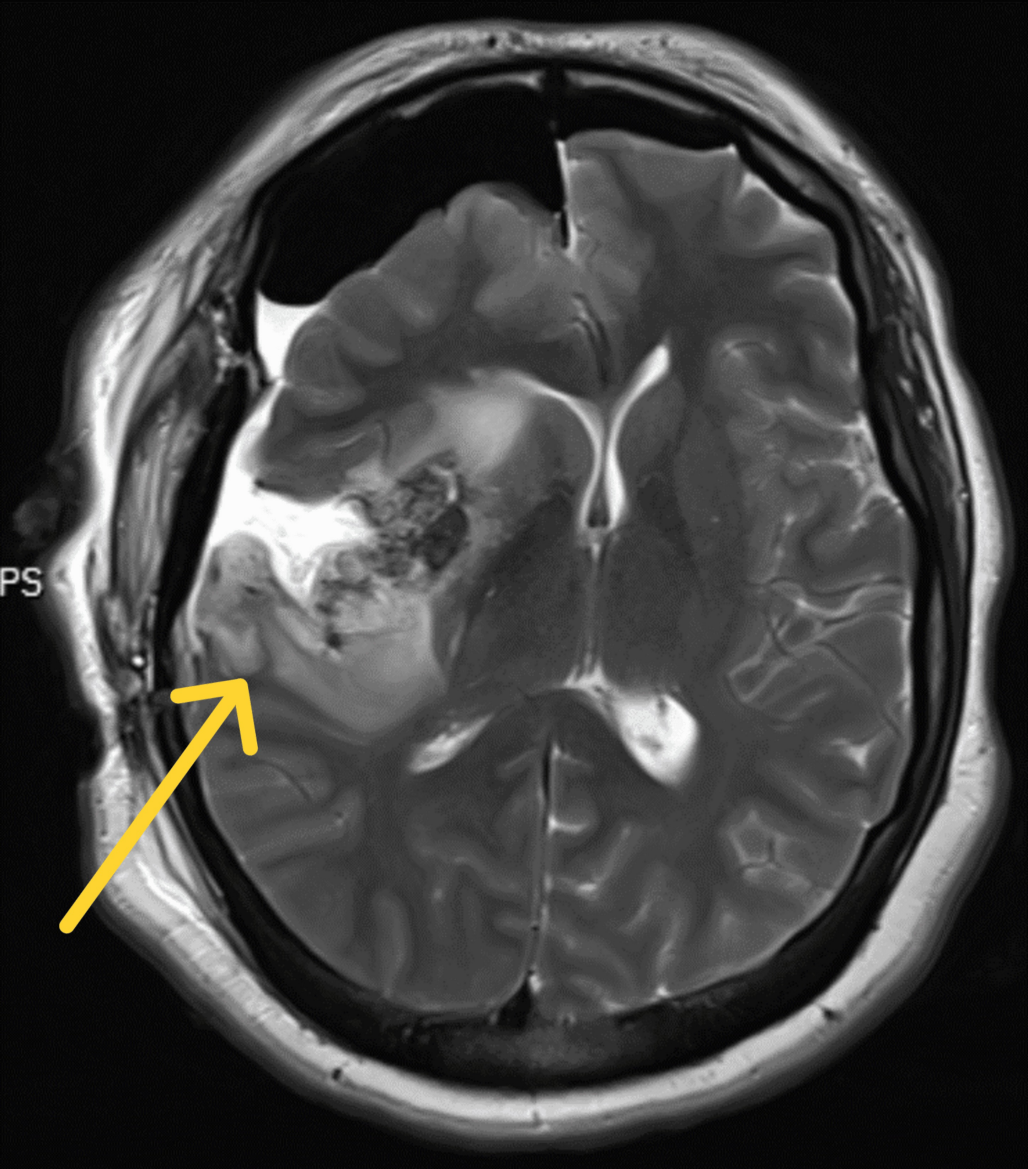
Postoperative MRI after second surgery (T2 axial view).

**Figure 4:**
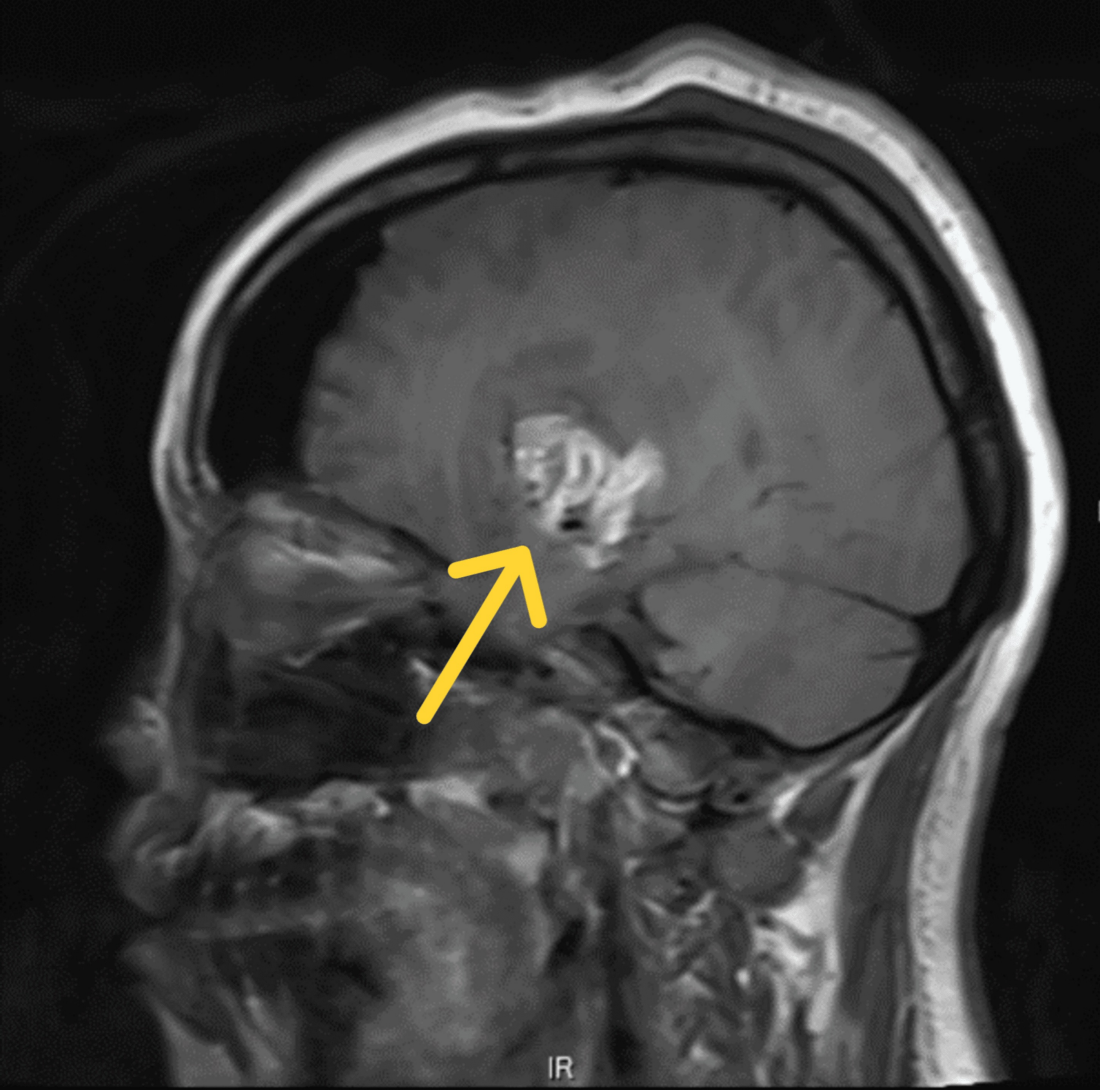
Postoperative MRI after second surgery (T1 with contrast, sagittal view).

After the second surgery, a postoperative MRI about 13 months after the first surgery showed a small residual tumor, indicating that near-total resection was achieved rather than complete removal. The images are shown in Figure 3 (T2 axial view) and Figure 4 (T1 with contrast, sagittal view).

Histopathology confirmed the diagnosis of an oligoastrocytoma, Grade II/IV (isocitrate dehydrogenase 1 (IDH1) mutant, Ki-67 proliferative index: 5%). This was based on histological features, which were standard practice before the 2016 WHO Classification. Testing showed the absence of 1p/19q co-deletion. At that time, this was not required to diagnose oligoastrocytomas. According to the current classification, a tumor without 1p/19q co-deletion would be reclassified as an astrocytoma, IDH-mutant. This reflects the shift to a more precise, molecular-based classification system.

Progression and complications

Follow-up MRI approximately 16 months after the initial surgery revealed multiple new enhancing nodules along the lateral and inferior aspects of the surgical cavity (1.5 × 1.2 cm), another one (0.8 × 0.5 cm) on the posterior part of the surgical cavity, and a 1.8 × 1 cm lesion over the temporal convexity. The patient did not receive radiotherapy or chemotherapy following the initial surgery due to the diagnosis of Grade II oligoastrocytoma at that time, which typically does not warrant immediate adjuvant therapy.

This case is particularly notable because the patient’s disease course spanned three iterations of the WHO CNS tumor classification (2004, 2016, and 2021). Under the 2004 WHO classification, the initial diagnosis of oligoastrocytoma was based on histopathological features without molecular markers. The introduction of molecular testing in 2016 and further refinements in 2021 emphasize the evolving understanding of such tumors.

Third surgery

The patient presented approximately 18 months after the initial diagnosis with complaints of headaches and uncontrolled focal seizures. Imaging revealed a 4.5 × 2.5 × 4 cm intracerebral hemorrhage and rapid tumor progression with evidence of heterogeneous enhancement.

**Figure 5:**
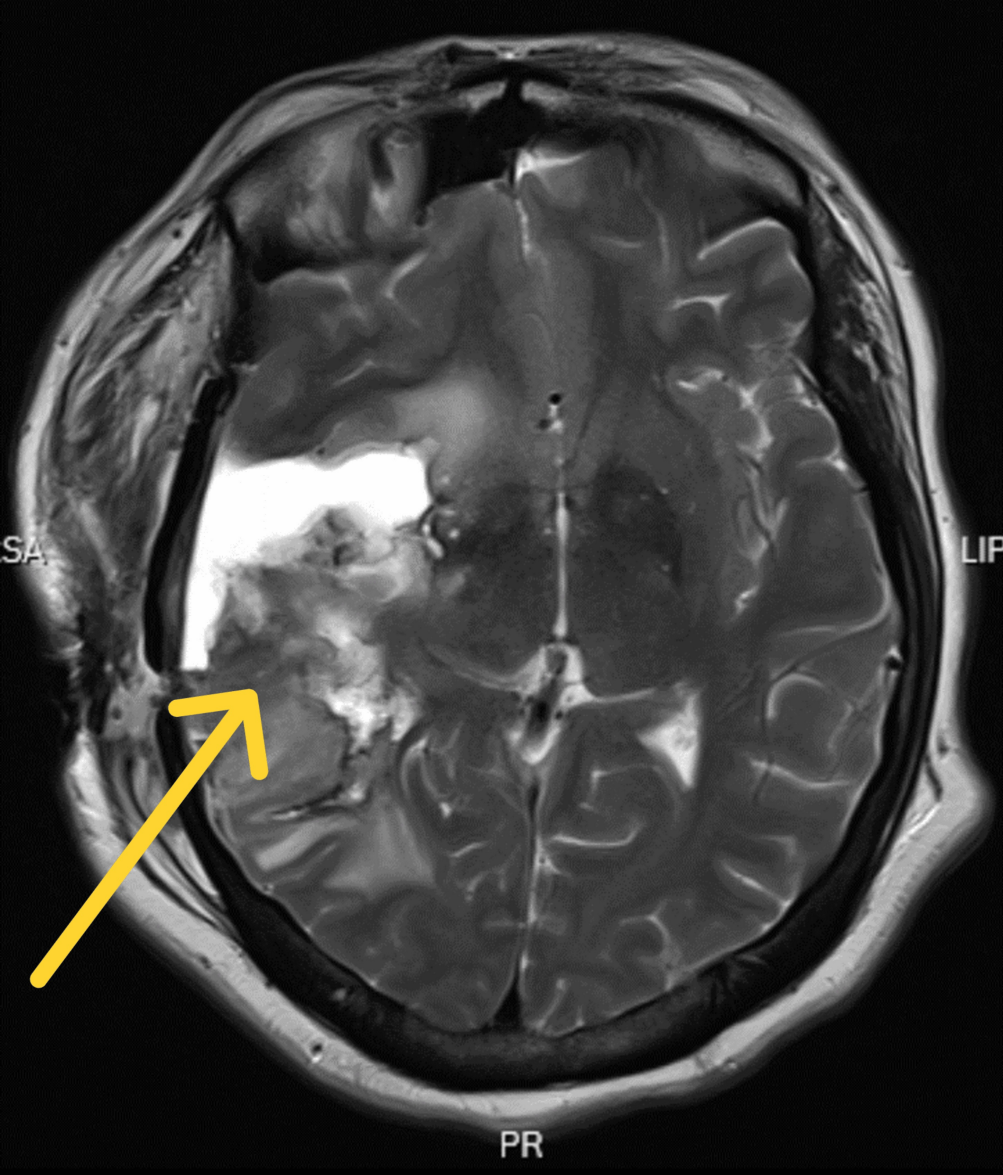
Postoperative MRI (T2 axial view) following redo craniotomy and tumor resection.

**Figure 6:**
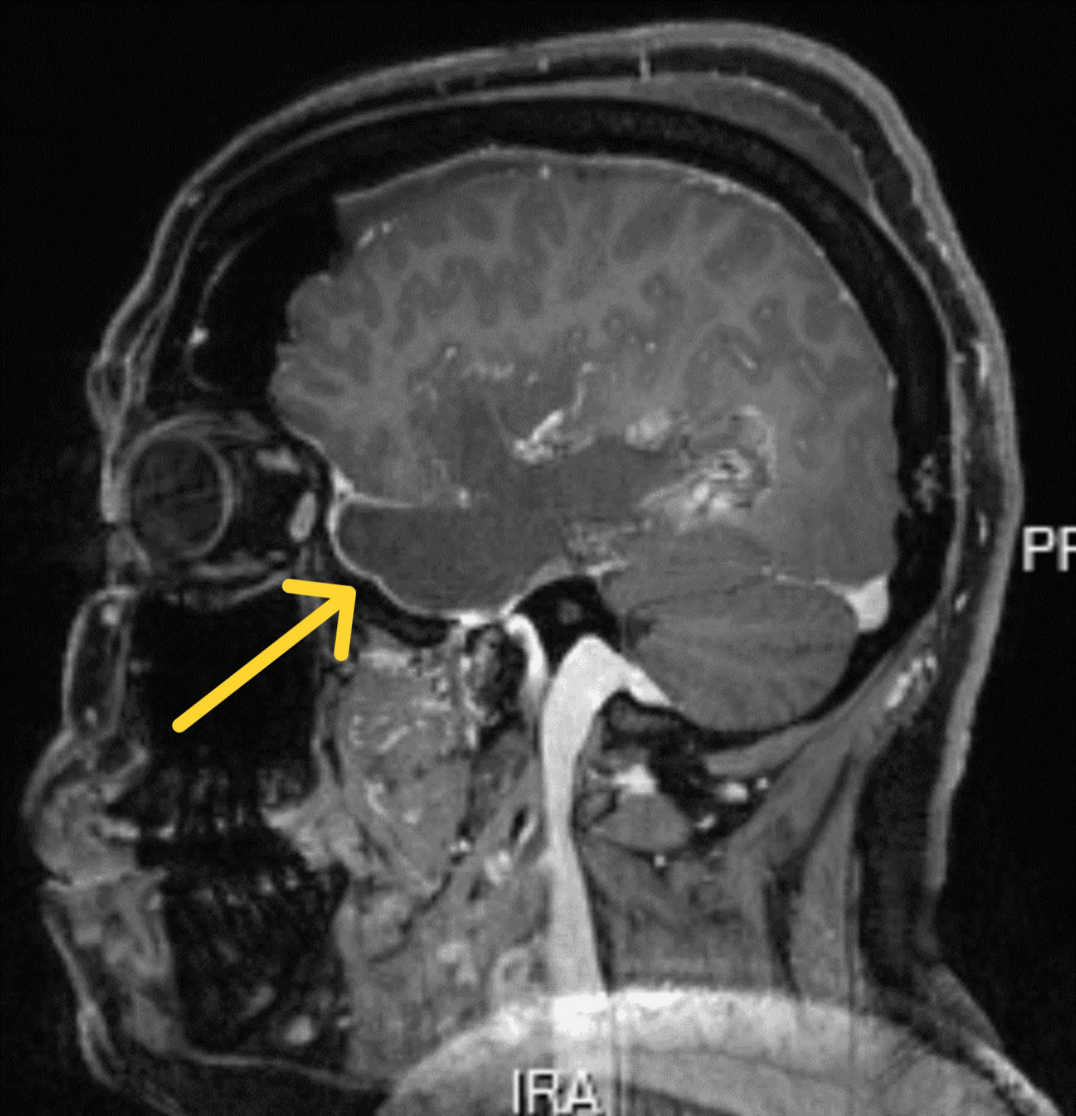
Postoperative MRI (T1 with contrast, axial view) following redo craniotomy and tumor resection.

Approximately 19 months after the initial diagnosis, a redo craniotomy and resection of the brain tumor were performed. A postoperative MRI showed a small residual tumor, as demonstrated in Figure 5 (T2 axial view) and Figure 6 (T1 with contrast, axial view), with imaging sequences taken at the same level as previous figures for consistency and objective comparison.

Pathology suggested the diagnosis of glioblastoma multiforme (according to the standard World Federation of Neurological Societies (WFNS) CNS tumor classification criteria at that time) with glial fibrillary acidic protein (GFAP), P53, and IDH positivity.

Concomitant radiotherapy and chemotherapy

Chemotherapy (temozolomide 75 mg/m (165 mg) and radiotherapy 1.5 grays in 30 fractions over six weeks) were planned; however, after the fourth radiotherapy session, she suffered increasing headache, and a CT scan of the head revealed right-side subacute subdural hematoma, which needed burr-hole evacuation. While radiotherapy and temozolomide are not direct causes of subdural hematomas, their side effects, such as thrombocytopenia, radiation-induced vascular fragility, and coagulopathy, can increase the risk of bleeding. In this case, the combination of recent radiotherapy, temozolomide-induced thrombocytopenia, underlying tumor progression, and prior surgical history likely contributed to the development of the subacute subdural hematoma. Following the hematoma drainage, antitumor therapy was halted to allow the patient to recover and to minimize the risk of further complications, such as recurrent bleeding or delayed healing.

A follow-up MRI approximately 20 months after the initial diagnosis revealed further tumor progression, showing both solid and cystic components.

Metastatic spread and subsequent management

**Figure 7:**
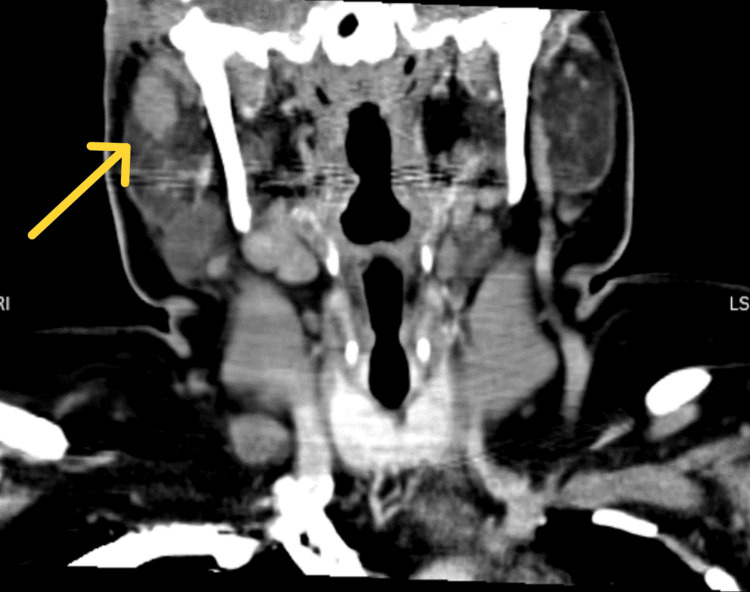
CT of the head and neck (coronal view) showing involvement of the parotid gland.

**Figure 8:**
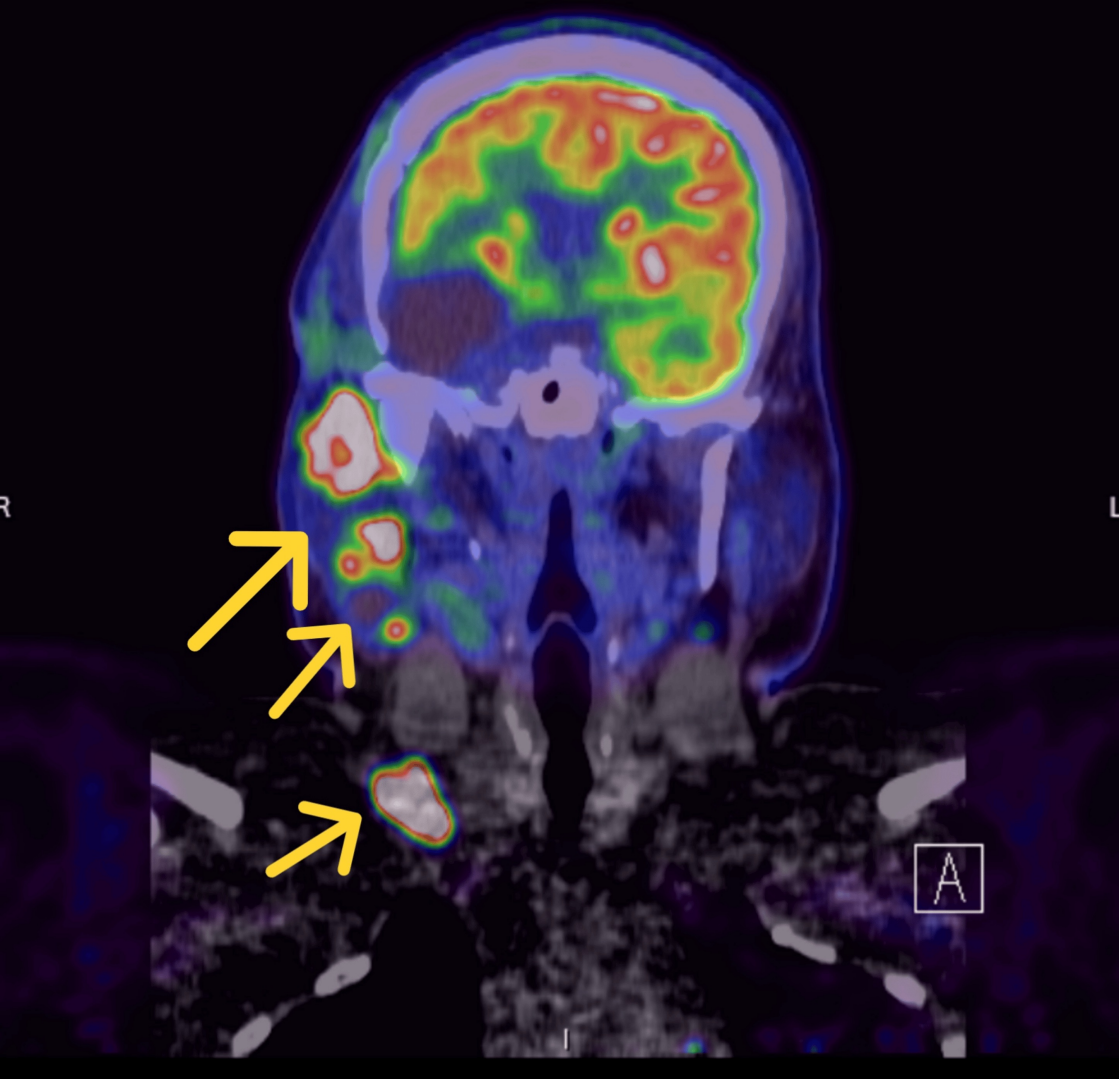
PET scan (coronal view) revealing metabolic activity consistent with tumor progression and metastatic involvement of the parotid gland, cervical and supraclavicular lymph nodes.

At approximately 23 months after the initial diagnosis, the patient presented with neck swelling and facial palsy. Imaging, including CT head and neck and a positron emission tomography (PET) scan, confirmed the suspicion of extracranial metastasis, as shown in Figures 7, 8. These images illustrate the extent of metastatic spread.

A Tru-cut needle biopsy revealed tumor cells consistent with malignancy. A neck dissection approximately 25 months after the initial presentation confirmed metastatic lesions, including a large mass occupying two-thirds of the parotid gland. The mass was embedding the facial nerve, invading the skin of the internal auditory canal, and infiltrating the sternocleidomastoid muscle. Pathology confirmed metastatic spread to the cervical lymph nodes, supraclavicular lymph nodes, and parotid gland, which is consistent with glioblastoma multiforme, with a Ki-67 proliferative index of 85%.

**Figure 9:**
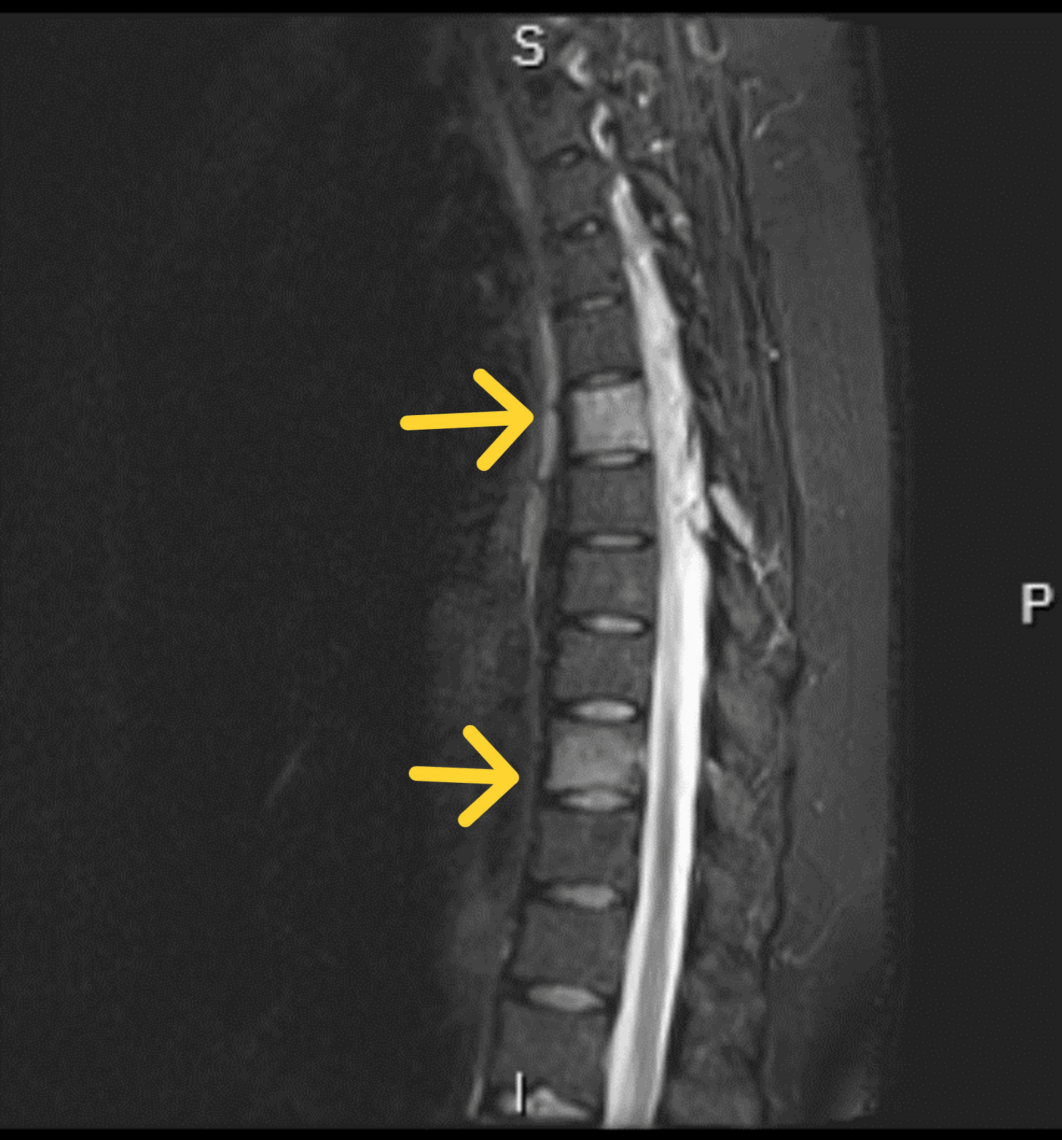
Sagittal MRI of the thoracic spine (T2-weighted).

**Figure 10:**
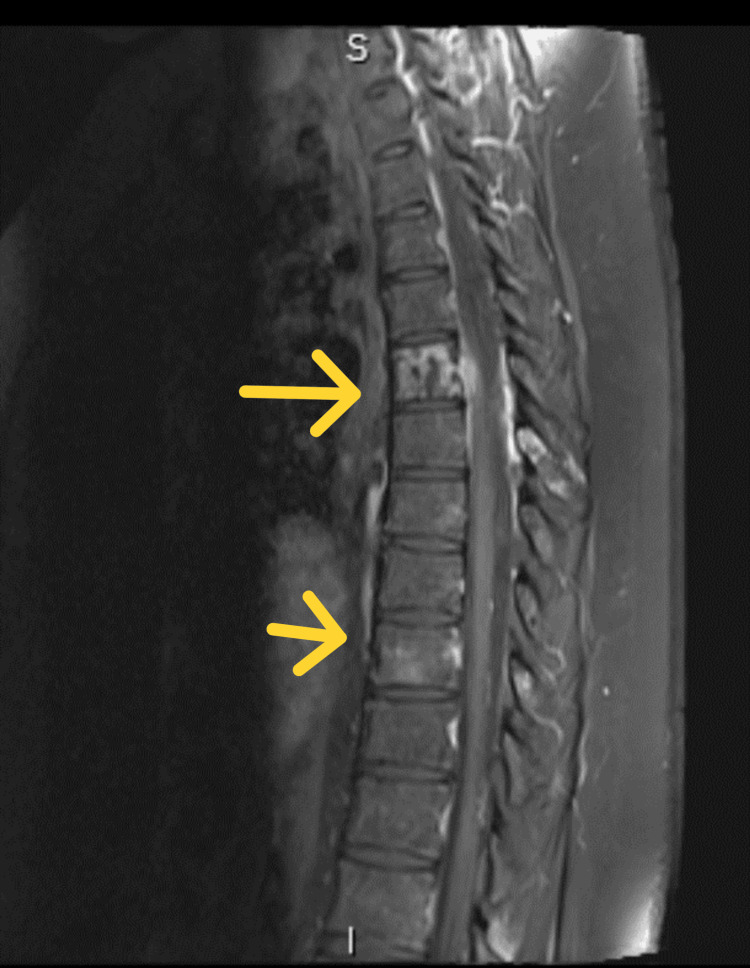
Sagittal MRI of the thoracic spine (T1 with contrast).

The patient subsequently complained of back pain. A spinal MRI approximately 27 months after the initial presentation revealed an epidural-enhancing lesion extending from T4 to T7. The patient received palliative radiotherapy with a dose of 30 Gy in 10 fractions to alleviate symptoms. The images, shown in Figure 9 (sagittal MRI of the thoracic spine, T2-weighted) and Figure 10 (sagittal MRI of the thoracic spine, T1 with contrast), were taken at consistent levels and sequences for objective comparison.

The patient was started on second-line chemotherapy with irinotecan and cisplatin. Bevacizumab was not included in the regimen due to the patient's history of a subdural hematoma, as the risk of further complications, such as bleeding, was a significant concern. The decision was made based on clinical judgment, prioritizing the patient’s safety and minimizing potential side effects while managing disease progression. Unfortunately, the disease progressed rapidly, with increasing tumor size in the right parotid area and neck regions. Her condition deteriorated progressively, and she passed away approximately 28 months after her initial presentation.

Histopathology

**Figure 11:**
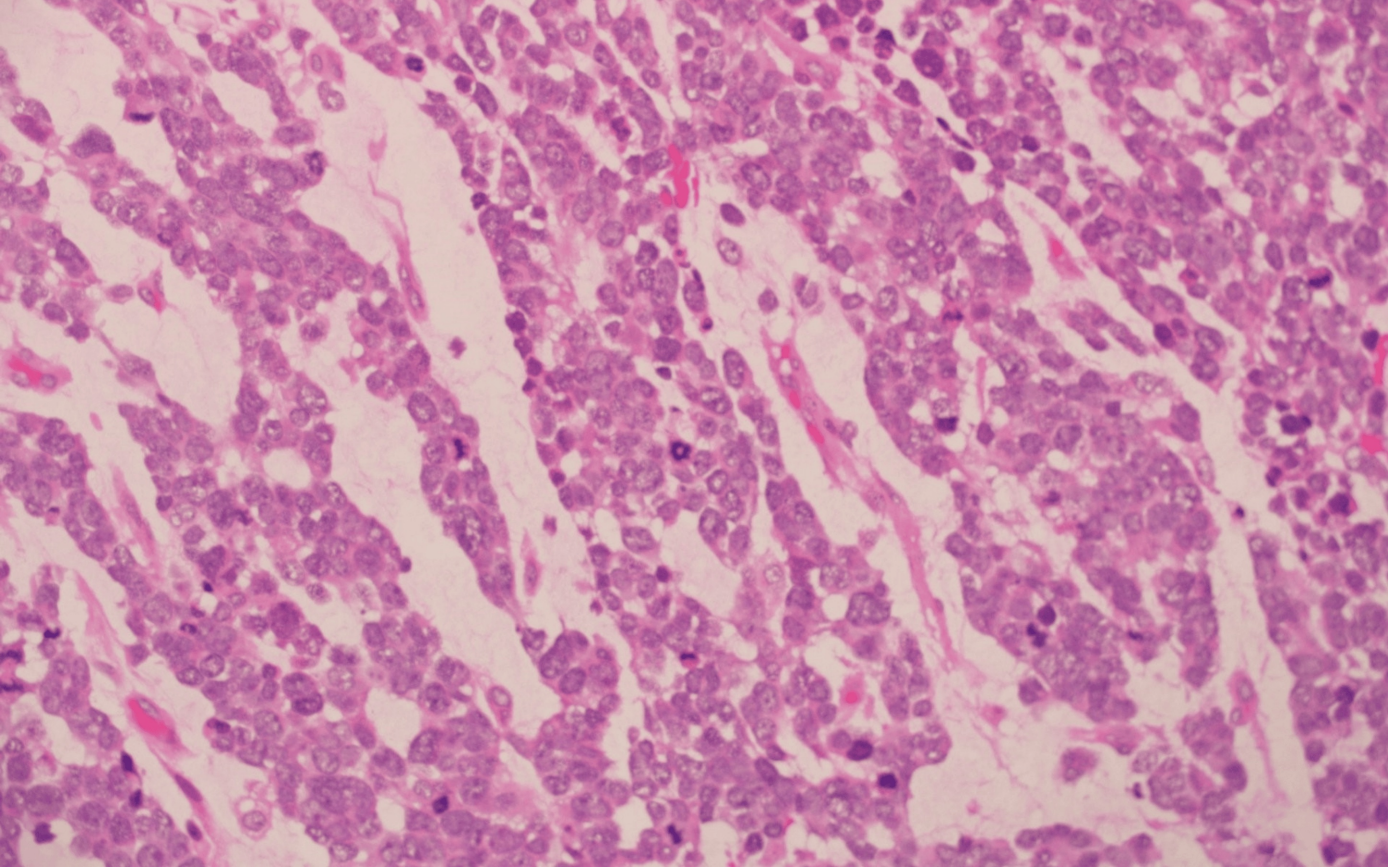
Tumor section showing hematoxylin and eosin (H&E) staining at 40x magnification.

**Figure 12:**
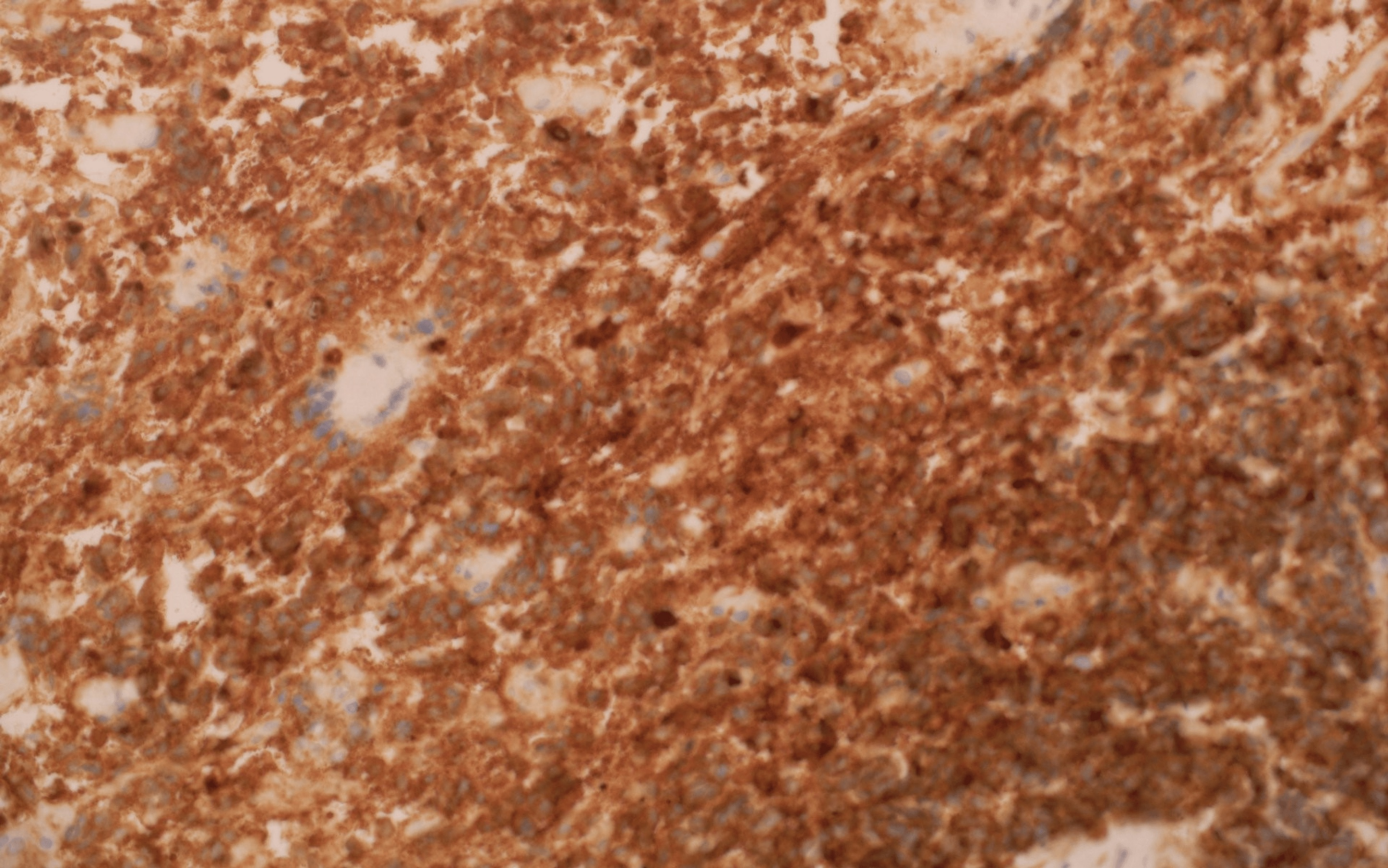
Tumor section showing glial fibrillary acidic protein (GFAP) staining at 40x magnification.

**Figure 13:**
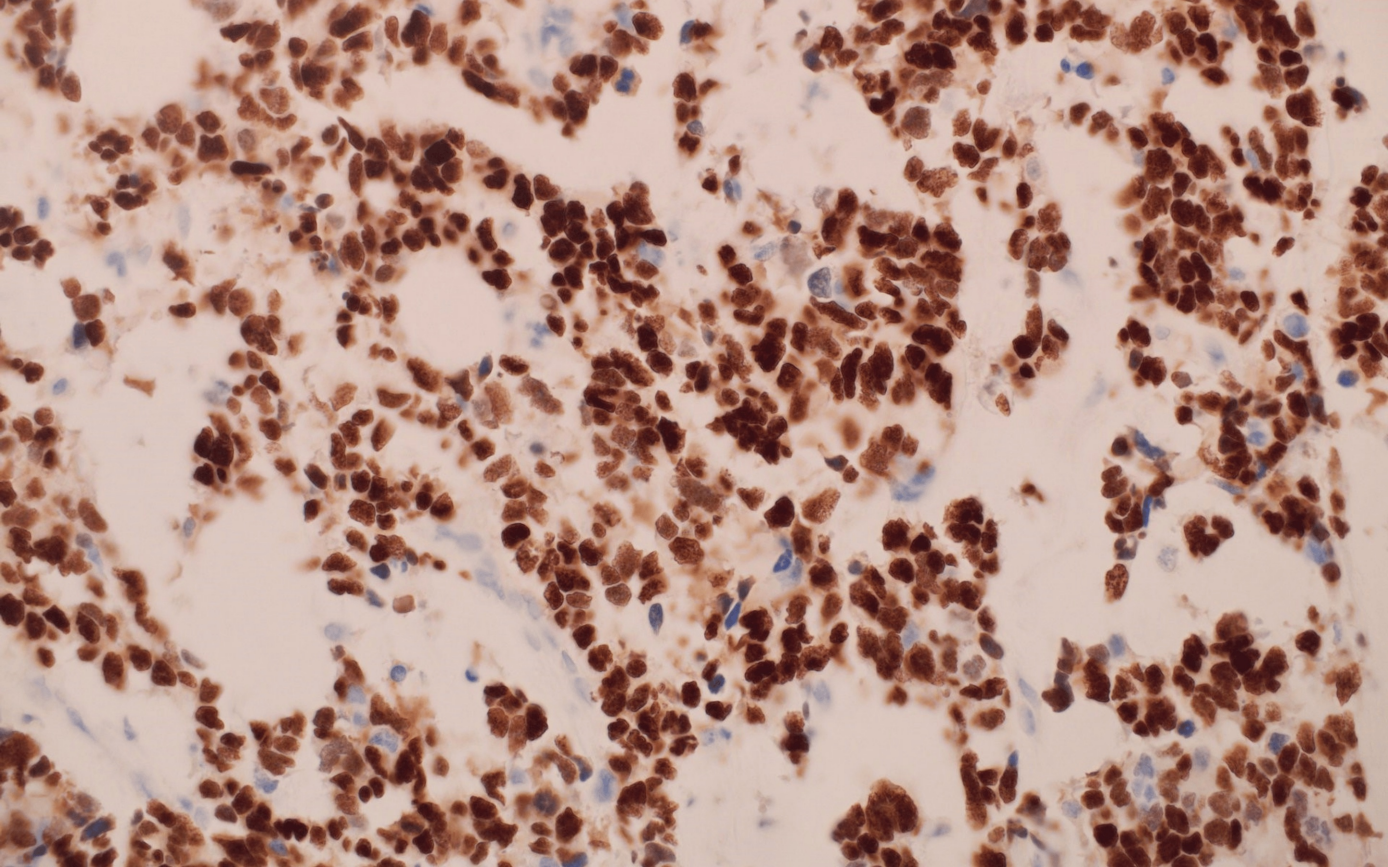
Tumor section showing oligodendrocyte transcription factor 2 (Olig2) staining at 100x magnification.

Histopathological and immunohistochemical analysis of the parotid gland tissue revealed definitive features of metastatic glioblastoma multiforme (GBM), providing crucial insights into the tumor’s high-grade malignancy and astrocytic origin. Figures 11, 12, 13 demonstrate the infiltration of the parotid gland by metastatic GBM, as shown by hematoxylin and eosin (H&E) staining (Figure 11), glial fibrillary acidic protein (GFAP) staining (Figure 12), and oligodendrocyte transcription factor 2 (Olig2) staining (Figure 13). While Olig2 is commonly associated with gliomas, including astrocytic tumors, it is not specific to oligodendrogliomas, contrary to common misconceptions. This combination of markers highlights the astrocytic origin of the metastatic lesions.

Figure 11, stained with H&E at 40x magnification, reveals the disruption of the normal glandular architecture by infiltrating tumor cells. These cells are characterized by a high nuclear-cytoplasmic ratio, pleomorphic nuclei, and areas of necrosis, indicative of high-grade malignancy.

Figure 12, stained with GFAP at 40x magnification, demonstrates intense and widespread brown coloration, confirming the glial lineage of the tumor cells within the parotid gland. This strongly supports astrocytic differentiation, consistent with glioblastoma metastasis when correlated with molecular findings and other histological features.

Figure 13, stained with Olig2 at 100x magnification, shows a uniform and extensive pattern of nuclear positivity, supporting the glial lineage of the tumor. However, fluorescence in situ hybridization (FISH) analysis revealed no 1p/19q co-deletion, excluding the possibility of oligodendroglioma. Olig2 positivity, while commonly seen in gliomas, is not exclusive to any specific glioma subtype.

The combination of histological, immunohistochemical, and molecular findings, including IDH1, R132H mutation, MYC amplification, and the absence of 1p/19q co-deletion, aligns with the diagnosis of astrocytoma, IDH-mutant, WHO Grade 4, based on the 2021 WHO classification. While the H&E findings highlight features of high-grade malignancy, the absence of earlier histological comparisons limits definitive correlation to glial origin solely based on this preparation.

Molecular testing and reclassification as of 2021 WHO Classification criteria

In 2024, the old saved surgical specimens taken from the neck were reviewed. Repeated molecular studies revealed the following results: MYC amplification on copy number variation (CNV), IDH1 mutation (R132H), PIK3CA mutation (E545K), RB1 mutation (Y529), and ATRX mutation (Q1319). The results were negative for a 1p36 deletion but showed added signals and were similarly negative for a 19q13 deletion with added signals.

These histological and immunohistochemical findings confirm the diagnosis of metastatic glioblastoma multiforme (GBM) in the parotid gland, based on the classification used at the time of the patient’s initial diagnosis. 

## Discussion

The patient was initially diagnosed in 2013 with a low-grade glioma that progressed to a high-grade glioma. At the time, the tumor was classified as glioblastoma multiforme (based on the then-valid WFNS classification). The patient later developed extracranial metastasis to the neck, confirmed as metastatic glioblastoma.

In preparation for publication years later, diagnostic challenges emerged due to the tumor classification updates introduced in the 2016 and 2021 WHO CNS tumor classifications. A review of saved surgical specimens from the patient’s last surgery was performed with the assistance of a senior neuropathologist. Repeated molecular testing, aligned with the 2021 WHO Classification of CNS tumors, revealed the presence of an IDH1 R132H mutation and the absence of 1p/19q co-deletion, leading to the reclassification of the tumor as astrocytoma, IDH-mutant, WHO Grade 4. This updated diagnosis highlights the histological features of glioblastoma multiforme but reflects its molecular distinction as an IDH-mutant astrocytic tumor.

Glioblastoma is an extremely aggressive malignant primary brain tumor that typically limits patient survival to about 15 months, even if it is treated with aggressive measures like surgery to remove as much of the tumor as possible, followed by radiation therapy and chemotherapy in certain cases [7]. Combined with its aggressive course, it results in an exceptionally low incidence of extracranial metastases, estimated to occur in only 0.2 to 2.7% of cases [8]. This rarity is partly due to the generally short survival time of patients, which limits the malignancy's opportunity to spread beyond the brain. 

The spread of glioblastoma cells to areas outside the brain is thought to occur through several mechanisms. One hypothesis suggests tumor cells may infiltrate extracranial blood vessels and glymphatic systems during surgical procedures [9]. Additionally, the formation of new blood vessels in the area operated on, particularly in the dura mater, might help the dissemination of these malignant cells. Another potential pathway for glioblastoma cells to spread extracranially is through the cerebrospinal fluid, leading to what is known as drop metastases, which did occur in this patient [10]. 

This process can lead to metastasis at various extracranial sites. In some cases, tumor cells can spread locally near surgical incisions due to disruption of the blood-brain barrier. Leptomeningeal dissemination can result in spinal metastases, while ventricular-peritoneal shunts have been documented as a pathway for abdominal spread. Tumor seeding along biopsy tracts has also been reported. Although rare, glioblastoma has metastasized to distant organs such as the lungs, pleura, liver, and bone marrow in documented cases [11]. In this case, the patient developed extracranial metastases to the neck and parotid gland. This rare occurrence highlights the aggressive behavior and dissemination potential of glioblastoma, even when primary treatment is focused on controlling intracranial disease.

The parotid gland, the largest salivary gland, is a highly unusual site for glioblastoma metastases. In most cases, metastases to the parotid arise from primary head and neck tumors, typically through lymphatic spread or, less commonly, hematogenous dissemination [12]. In this patient, glioblastoma cells likely spread to the parotid gland through a combination of factors. Tumor seeding during prior surgical procedures remains a plausible explanation, as glioblastoma cells are known to disseminate along surgical tracts [13]. Additionally, vascular invasion may have facilitated distant metastasis, given the tumor’s highly aggressive behavior. The role of thin-walled blood vessels in the dura mater as a potential route, as proposed by Willis, is another hypothesis, though this alone does not fully explain the spread to extracranial sites such as the parotid gland [14].

In this case, the spread of the tumor likely involved multiple mechanisms. The patient underwent several surgeries, including craniotomies, which could have created opportunities for tumor cells to spread beyond their original site. Surgical manipulation may have disrupted surrounding tissues, allowing tumor cells to reach extracranial areas. The tumor’s progression from a low-grade astrocytoma to an aggressive astrocytoma, IDH-mutant, WHO Grade 4, despite treatment, reflects its highly aggressive nature. This rapid transformation likely contributed to the spread, as faster-growing tumors often invade nearby tissues and have more chances to extend to distant sites.

The involvement of the cervical lymph nodes and the parotid gland points to a possible lymphatic route of spread. While gliomas do not typically metastasize through the lymphatic system, the aggressive behavior of this tumor makes it a plausible explanation in this case. Another possible pathway involves the glymphatic system, which is responsible for clearing waste and interstitial fluid within the CNS. This system may have facilitated tumor migration through cerebrospinal fluid (CSF), potentially explaining the drop in metastases seen in the spine. Hematogenous spread is also a likely mechanism, considering the tumor’s aggressive nature and ability to infiltrate blood vessels. This could have contributed to the distant involvement of the parotid gland and spine.

Additionally, the tumor exhibited extensive local invasion, affecting the skin, external auditory canal, and deep facial structures. While such invasion is rare in gliomas, it can occur in advanced stages. Taken together, the local invasion, potential lymphatic and glymphatic involvement, and hematogenous spread reflect the rare and complex metastatic behavior of high-grade gliomas, as seen in this patient.

Diagnosing glioblastoma with parotid metastases poses significant challenges, even in patients with a known history of the disease. The rarity of this presentation, combined with the potential for other malignancies in the parotid gland, adds complexity to the diagnostic process. Imaging techniques, such as MRI or CT scans, can help evaluate the lesion and provide important clues, but they cannot confirm the tumor’s origin. A definitive diagnosis requires examining tissue samples, which can confirm whether the lesion is metastatic glioblastoma. Fine needle aspiration cytology (FNAC) may provide an initial evaluation, but it is generally insufficient to make a definitive diagnosis in such cases. A thorough analysis of tissue, often supported by immunohistochemistry or molecular studies, is usually required to confirm the tumor type and its origin. This case highlights the unique challenges of diagnosing such rare metastases and underscores the importance of a comprehensive diagnostic approach.

Treating glioblastoma with parotid metastases poses significant challenges due to the rarity of this condition. While treatment generally includes surgery, radiotherapy, and chemotherapy, the effectiveness of these approaches for extracranial metastases is uncertain. Decisions regarding treatment often depend on the patient’s overall condition and the extent of metastasis, with some patients receiving aggressive therapy and others opting for palliative care.

Despite these efforts, the prognosis remains poor, highlighting the need for further exploration of therapeutic strategies for this rare manifestation. Table 1 summarizes reported cases of glioblastoma with parotid gland metastases, focusing on treatment approaches and survival outcomes.

**Table 1:** Literature review and similar cases in the literature. This table includes cases of glioblastoma, with all tumors retrospectively classified according to the WHO 2021 classification for central nervous system (CNS) tumors. Although the studies were published across different years, the classification has been standardized to reflect current diagnostic criteria, ensuring consistency in terminology and understanding

Primary Author	Age/Sex	Primary Lesion	Site of Metastasis	Treatment Provided	Survival
Romero-Rojas et al. [15]	26/Male	Glioblastoma	Parotid gland, neck lymph nodes	Temozolomide, radiotherapy	2 years
Swinnen et al. [16]	56/Female	Glioblastoma	Parotid gland, cervical lymph nodes, lungs	Surgical resection, radiation therapy	18 months
Alhoulaiby et al. [17]	56/Male	Glioblastoma	Parotid gland	Surgical resection, temozolomide radiation therapy	1.5 years
Kraft et al. [18]	58/Male	Glioblastoma	Parotid gland	Surgical resection, radiation therapy	15 months
Ogungbo et al. [19]	49/Male	Glioblastoma	Parotid gland	Surgical resection, chemotherapy, radiation therapy	24 months
Alsubaie et al. [20]	59/Male	Glioblastoma	Parotid gland	Surgical resection, chemotherapy, radiation therapy	20 months
Coca-Pelaz et al. [21]	43/Male	Glioblastoma	Cervical lymph nodes parotid gland	Unknown	Unknown
Waite et al. [22]	40/Male	Glioblastoma	Cervical lymph nodes parotid gland	Macroscopic total removal + frontal lobectomy + postoperative radiation therapy (60 Gy)	2 months
Park et al. [23]	25/Female	Glioblastoma	Parotid gland and skin	Subtotal resection + external beam radiation therapy to 59.4 Gy	6 months
Kühn et al. [24]	58/Female	Glioblastoma	Cervical lymph nodes parotid gland	Macroscopic total removal + temporal lobectomy + later radiation therapy	Unknown
Taha et al. [25]	33/Male	Glioblastoma	Cervical lymph nodes parotid gland	Postoperative radiation therapy (60 Gy) + chemotherapy	3 months
Taskapılıoglu et al. [26]	30/Female	Glioblastoma	Parotid gland, bones, lymph nodes	Postoperative radiation therapy + palliative chemotherapy (temozolomide )	6 months
Liu et al. [05]	46/Male	Glioblastoma	Parotid gland, lungs	Surgical resection, chemotherapy temozolomide radiation therapy	20 months
Swinnen et al. [16]	56/Male	Glioblastoma	Parotid gland, lungs	Postoperative radiation therapy + palliative chemotherapy (temozolomide + axitinib + avelumab)	11 months
Kraft et al. [18]	58/Male	Glioblastoma	Parotid gland	Postoperative radiation therapy + palliative chemotherapy (temozolomide)	2 months
Ogungbo et al. [19]	44/Female	Glioblastoma	Parotid gland	Postoperative radiation therapy (total 30 Gy) + chemotherapy (CCNU + procarbazine)	11 months

In presenting this case, we acknowledge that some radiological images from earlier stages of the disease were not retrievable due to a transition to a new electronic health record system. Despite this, we have provided a detailed account of the patient’s diagnostic and clinical journey, supported by documented imaging findings from later stages, histopathologic analyses, and molecular studies. These comprehensive descriptions illustrate the progression and rare metastatic behavior of glioblastoma, offering valuable insights into this unusual presentation. The inclusion of histological and immunohistochemical studies from surgical specimens further substantiates the diagnosis, providing a well-rounded perspective on this case.

## Conclusions

This case highlights the infrequent occurrence of high-grade glioma, classified as astrocytoma, IDH-mutant, WHO Grade 4, metastasizing to the parotid gland, cervical lymph nodes, and supraclavicular lymph nodes, as confirmed by PET-CT. While high-grade gliomas typically remain confined to the central nervous system due to their aggressive intracranial progression, this case demonstrates an atypical progression with extracranial metastases. The involvement of the parotid gland and lymph nodes emphasizes the importance of considering extracranial spread in high-grade gliomas presenting with unusual clinical features. Despite aggressive treatment, including surgery, chemotherapy, and radiotherapy, the disease progressed relentlessly. This underscores the need for heightened clinical awareness of such rare metastatic patterns and further research into the mechanisms and management of extracranial metastases in high-grade gliomas.
